# PCR Detection, Genotyping, and Differentiation of *Toxoplasma gondii* from *Hammondia hammondi* Excreted in the Feces of Cats in Poland Between 2020 and 2024

**DOI:** 10.3390/pathogens14050444

**Published:** 2025-04-30

**Authors:** Dawid Jańczak, Rafał Stryjek, Aleksandra Kornelia Maj, Jakub Olszewski, Olga Szaluś-Jordanow

**Affiliations:** 1 Department of Infectious and Invasive Diseases and Veterinary Administration, Institute of Veterinary Medicine, Faculty of Biological and Veterinary Sciences, Nicolaus Copernicus University, 87-100 Toruń, Poland; 2 Institute of Psychology, Polish Academy of Sciences, 00-378 Warsaw, Poland; rstryjek@wp.pl; 3 Animallab Veterinary Laboratory, 03-430 Warsaw, Poland; aleksandra.kornelia.maj@gmail.com (A.K.M.); jakubolszewski2424@gmail.com (J.O.); 4 Department of Small Animal Diseases with Clinic, Institute of Veterinary Medicine, Warsaw University of Life Sciences-SGGW, 02-776 Warsaw, Poland

**Keywords:** *Toxoplasma gondii*, multilocus genotyping, feces, cats, Poland

## Abstract

Toxoplasmosis, caused by *Toxoplasma gondii*, is a widespread parasitic infection affecting humans and animals. The genetic diversity of *T. gondii* varies across regions, with type I, II, and III strains predominantly circulating in Europe and North America. This study genotyped 67 (78.8%) *T. gondii* DNA isolates from cats using nested and multilocus PCR-RFLP, identifying type I, genotype #10 (ToxoDB#10), for the first time in Poland. The other 18 (21.2%) stool samples containing *T. gondii*-like oocysts were confirmed as *Hammondia hammondi.* Comparative analysis with data from other countries highlights notable regional differences in genotype prevalence. The high occurrence of genotype 3 (ToxoDB#3) in central Europe may be linked to its presence in wild rodents and insectivores, key reservoirs in the parasite’s life cycle. Additionally, genetic analysis of meat products and livestock indicates a potential transmission pathway to felines through raw or undercooked meat consumption. These findings contribute to a better understanding of *T. gondii* epidemiology and its implications for public health and veterinary medicine.

## 1. Introduction

*Toxoplasma gondii* is an intracellular protozoan parasite capable of infecting all warm-blooded animals, in contrast to *Hammondia hammondi,* which can naturally infect only a limited range of animals like mice, rats, and felids [1,2,3]. House cats and other felids are the only animals that can excrete *T. gondii* and *H. hammondi* oocysts into the environment through their feces. *T. gondii* oocysts become infective after sporulating for 3 to 5 days and can remain viable in water and soil for many months [4,5,6]. However, the sporulation period can be as short as one day under optimal environmental conditions, such as those found in cat litter [7]. Approximately one-third of the global human population is infected with *T. gondii*; however, clinical symptoms, such as fever, ocular inflammation, and lymphadenopathy, occur in fewer than 20% of infected individuals [8,9]. Toxoplasmosis poses a significant challenge in immunocompromised patients, where generalized infection may develop, leading to neurological, lymphatic, or ocular complications [10]. The primary sources of *T. gondii* infection in humans include raw meat, raw milk, or mature cheese containing viable tachyzoites or bradyzoites of the parasite [11,12]. Cats and other felids become infected after consuming raw meat or tissues from any warm-blooded animal that can act as an intermediate host [13]. In young cats, during their first infection, clinical signs such as diarrhea, lethargy, polydipsia, jaundice, dyspnea, and fever may be observed [14]. Infected cats can shed between 3 and 8 million oocysts during the shedding period, although in some cases, no oocysts are excreted at all [15]. Zulpo et al. confirmed that the shedding of *T. gondii* oocysts may remain elevated in experimentally re-infected cats even years after the primary infection. Approximately 10% and 71% of cats re-infected with different strains were found to excrete oocysts following secondary and tertiary infections, respectively [16].

Dubey demonstrated a relationship between *T. gondii* infection and *Cystoisospora felis*. In cats initially infected with *T. gondii* and subsequently with *C. felis*, reactivation of toxoplasmosis and oocyst shedding could occur. However, when the order of infection was reversed, the local intestinal immunity induced by *C. felis* was sufficiently strong to prevent the shedding of *T. gondii* oocysts. Moreover, in adult cats, a reshedding may be induced by co-infection with other infectious diseases or long-term treatment with immunosuppressive drugs [17].

The oocysts of *T. gondii* and *Hammondia hammondi* are very similar in morphology, and the parasites themselves are closely related as tissue cyst-forming coccidians [18]. However, the bioassay procedure can differentiate them. *T. gondii* tissue cysts are more commonly found in neural tissue, whereas *H. hammondi* cysts predominantly localize in skeletal muscle. Additionally, tachyzoites and bradyzoites of *H. hammondii* are not infective after oral administration. Moreover, *H. hammondi* appears to be non-pathogenic and does not cause clinical disease in cats or any naturally infected hosts, including humans [6]. Molecular techniques based on the polymerase chain reaction (PCR) are the methods for differentiating *Hammondia*-like organisms from *T. gondii* oocysts [19,20]. Due to variations in virulence among *T. gondii* strains, multilocus PCR-RFLP (restriction fragment length polymorphism) genotyping plays a crucial role in determining the geographic distribution and genetic diversity of clonal lineages [21,22,23,24,25]. In this study, we present the results of *T. gondii* and *H. hammondi* oocyst differentiation using PCR methods and a molecular analysis of *T. gondii* strains isolated from feline feces collected in Poland between 2020 and 2024.

## 2. Materials and Methods

### 2.1. Sample Collection

**Figure 1:**
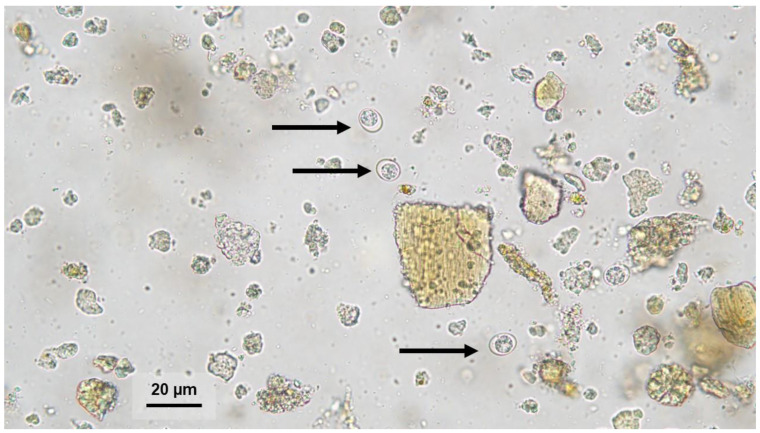
Oocyst (black arrows) morphologically resembling *Toxoplasma gondii* or *Hammondia hammondi* in a feline fecal sample detected by zinc sulfate flotation. Magnification 400×.

Between 2020 and 2024, a total of 61,648 feline fecal samples were examined using the zinc flotation method (ZnSO_4_, Specific Gravity = 1.31) and direct smear microscopy in a 0.9% saline solution. All stool samples were examined in a commercial veterinary laboratory as part of the diagnosis and prevention of gastrointestinal parasitic infestation. Stool consistency was evaluated in the laboratory prior to microscopic examination. Samples containing oocysts smaller than 15 μm in diameter were frozen for further molecular analysis (Figure 1). 

### 2.2. DNA Isolation and Molecular Analysis

DNA extraction was performed using the commercial Genomic Mini AX Stool kit (A&A Biotechnology, Gdańsk, Poland). The extracted DNA was suspended in 200 μL of elution buffer and stored at −20 °C for further analysis.

To differentiate *T. gondii* and *H. hammondii*, two different PCR tests were used [19,26]. Samples positive for *T. gondii* were subsequently analyzed by the nested and multiplex multilocus PCR-RFLP (Mn-PCR-RFLP) using various genetic markers: the B1 gene [21,27] and SAG1, 5′SAG2, 3′SAG2, alt.SAG2, SAG3, BTUB, GRA6, Apico, C22-8, C29-2, L358, and PK1 [8,24,28,29,30,31]. The primers and restriction enzymes applied in this study are presented in Table 1 and Table 2. Both PCR and nested PCR were carried out via a MultiGene optiMAX thermal cycler (Labnet International, Inc., Taoyuan, Taiwan) using a commercial StartWarm HS-PCR Mix kit (A&A Biotechnology, Gdańsk, Poland). The 530 bp products of B1 gene amplification were sequenced and analyzed using Chromas 2.6.6 (Technelysium Pty Ltd., South Brisbane, Australia) and MEGA version 7 software. To compare the obtained nucleotide sequences with the NCBI GenBank database, the Basic Local Alignment Search Tool (BLAST^®^, https://blast.ncbi.nlm.nih.gov/Blast.cgi; accessed on 3 December 2024) was employed. Amplification products from nested and Mn-PCR-RFLP reactions were visualized on a 1.2% agarose gel under ultraviolet light. Subsequently, Mn-PCR-RFLP products were digested with restriction enzymes in a 20 μL volume and separated on a 3.5% agarose gel. The obtained band patterns were compared with data from ToxoDB (https://toxodb.org/toxo/; accessed on 16 December 2024) and two research papers [32,33] to determine the *T. gondii* genotype (Table 3).

### 2.3. Statistical Analysis

Statistical analyses were conducted using IBM SPSS Statistics version 29.0 (Armonk, NY, USA) for the following variables: sex (female, male), age (in years), co-infection (e.g., presence of other intestinal parasites such as *Giardia duodenalis* or *Cystoisospora* spp.), stool consistency (formed, diarrhetic), and parasite genotype (*T. gondii* type I or II, *H. hammondi*). Only statistically significant results (*p* ≤ 0.05) are reported. Given that most variables were measured on a nominal scale and the age variable did not meet the assumption of normality, non-parametric tests were applied.

## 3. Results

**Figure 2:**
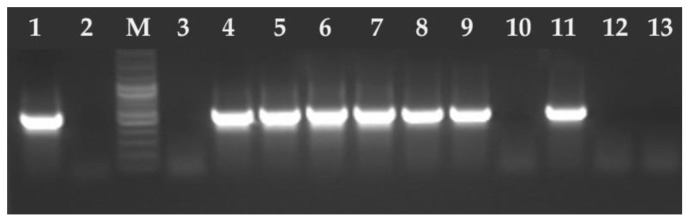
Electrophoresis results of the PCR products on a 2% agarose gel. The amplified PCR product 530 bp *T. gondii* B1 gene fragment. Lane 1—positive control RH strain of *T. gondii*, lane 2—negative control (ddH_2_O), M—marker (Marker 3, A&A Biotechnology, Gdańsk, Poland), lanes 3, 10, 12–13 negative samples, lanes 4–9 and 11 positive samples.

**Figure 3:**
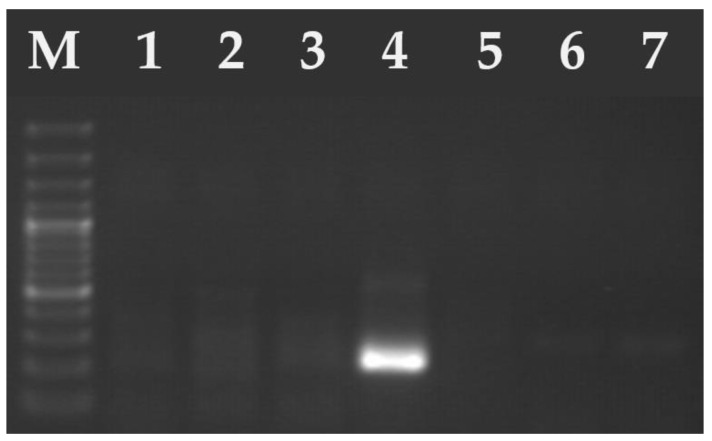
Electrophoresis results of the PCR products on a 2% agarose gel. The amplified PCR product ~240 bp of *H. hammondi* (lane 4—positive sample). Lanes 1–3 and 5–6 samples were negative for *H. hammondi*. M—marker (Marker 3, A&A Biotechnology, Gdańsk, Poland).

**Figure 4:**
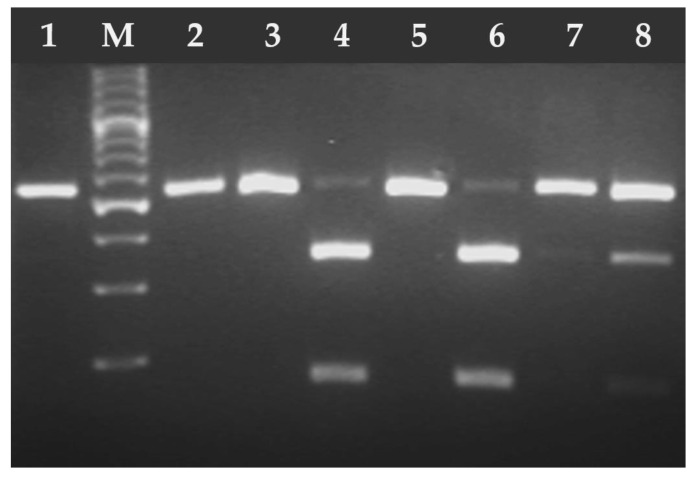
Electrophoresis results of the RFLP-PCR products of *T. gondii* B1 gene fragment digested with PmlI (Eco72) endonuclease. Lane 1 *T. gondii* RH strain (type I), lanes 2–8 *T. gondii* DNA isolates from feline feces; lanes 2–3, 5, 7 (type I), lanes 4, 6, and 8 (type II/III). M—marker (Marker 3, A&A Biotechnology, Gdańsk, Poland).

**Figure 5:**
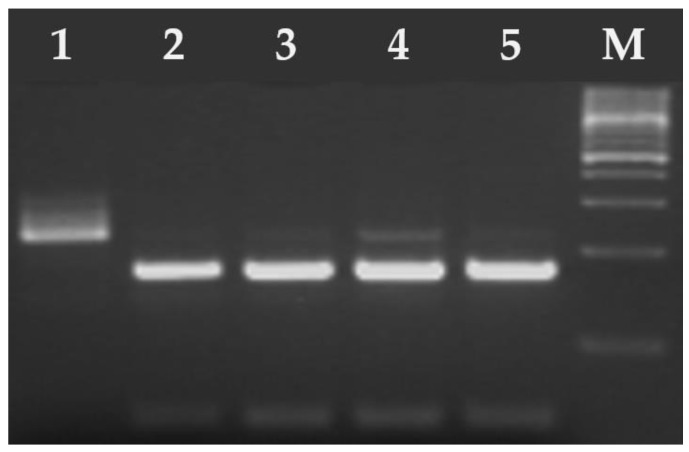
Electrophoresis results of the RFLP-PCR products of *T. gondii* SAG2-3′ digested with HhaI endonuclease. Lane 1 *T. gondii* RH strain (type I), lanes 2–5 *T. gondii* DNA isolates from feline feces (type II/III). M—marker (Marker 3, A&A Biotechnology, Gdańsk, Poland).

**Figure 6:**
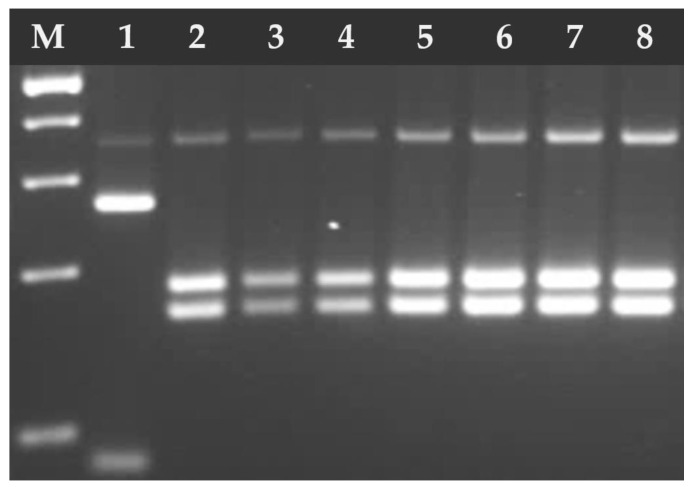
Electrophoresis results of the RFLP-PCR products of *T. gondii* GRA6 digested with MseI endonuclease. Lane 1 *T. gondii* RH strain (type I), lanes 2–8 *T. gondii* DNA isolates from feline feces (type II). M—marker (Marker 3, A&A Biotechnology, Gdańsk, Poland).

Between 2020 and 2024, oocysts smaller than 15 μm in diameter were found in 0.14% (85/61,648) of feline stool samples. The 67 (0.11%) samples were tested positive for *T. gondii* and 18 (0.03%) for *H. hammondi* (Figure 2 and Figure 3). No co-infection of *T. gondii* and *H. hammondi* was observed. The annual detection rates of *T. gondii* and *H. hammondi* are presented in Table 4. Among 67 *T. gondii* DNA isolates analyzed, 13 belong to clonal lineage I and 54 to lineage II (Figure 4, Figure 5 and Figure 6). Additional information on co-infections, stool consistency, sex, age of cats, and *T. gondii* strain types is provided in Table 5. Nested and Mn-PCR-RFLP enabled the identification of three *T. gondii* genotypes: ToxoDB#1 genotype (11.9%; 8/67), ToxoDB#3 (68.7%; 46/67), and ToxoDB#10 (19.4%; 13/67). All 13 genetic markers were successfully amplified, which can be explained by the large number of oocysts excreted in the cat feces (Table 3).

The statistical analyses were based on data obtained from 85 domestic cats (45 females and 40 males), aged approximately 2 months to 18 years. The overall mean age was 2.71 years (SD = 4.09); female cats had a mean age of 1.59 years (SD = 2.43), while males averaged 3.97 years (SD = 5.12).

A chi-square goodness-of-fit test revealed no significant difference in *T. gondii* prevalence between females (*n* = 36) and males (*n* = 31), χ^2^(1) = 0.37, *p* = 0.541. Among cats under 1 year of age, *T. gondii* (*n* = 45) was significantly more prevalent than *H. hammondi* (*n* = 12), χ^2^(1) = 19.11, *p* < 0.001. This trend was consistent across the full sample: *T. gondii* (*n* = 67) occurred significantly more frequently than *H. hammondi* (*n* = 18), χ^2^(1) = 28.25, *p* < 0.001. A chi-square test for independence indicated a significant association between age group and the occurrence of diarrhea, with younger cats (<1 year) exhibiting diarrhea more frequently than older individuals, χ^2^(1) = 7.11, *p* = 0.008, Cramer’s V = 0.317. Cats infected with *T. gondii* type I (ToxoDB#10) were significantly younger (mean rank = 20.54, *n* = 13) compared to those infected with type II (ToxoDB#1 and 3), (mean rank = 37.24, *n* = 54), as revealed by a Mann–Whitney U test, U = 176.00, Z = –2.78, *p* = 0.005, r = 0.34. The type of parasite was also significantly associated with the presence of diarrhea, which occurred more frequently in cats shedding *T. gondii* than in those shedding *H. hammondi*, χ^2^(1) = 13.08, *p* < 0.001, Cramer’s V = 0.424. However, there was no significant association between parasite type and the presence of co-infections, χ^2^(1) = 0.041, *p* = 0.839 (Table 4). The lack of detailed information regarding the cats’ origin, lifestyle, and diet limits the possibility of providing a more comprehensive context for the findings.

## 4. Discussion

Cats and other felids are the only mammals in which the sexual phase of the life cycle of *T. gondii* or *H. hammondi* occurs [2,34]. Domestic cats living in close proximity to humans are susceptible to *T. gondii* infection regardless of age, sex, or breed [35]. During the shedding period, cats can excrete over three million oocysts into the environment via feces [15].

However, the shedding of *T. gondii* oocysts typically precedes the development of specific antibodies, rendering serological tests ineffective for detecting active oocyst excretion [36]. Lappin et al. emphasize that diagnosing toxoplasmosis in cats through coproscopic examination is unreliable, due to both the brief duration of oocyst shedding and the low sensitivity of this diagnostic method [36]. The oocysts of *T. gondii* and *H. hammondi* are morphologically very similar, making them difficult to distinguish through routine microscopic examination [37]. However, certain features observable in histopathological analysis and pathophysiological differences following bioassays in mice, such as the infectivity of tachyzoites and bradyzoites or the ability for congenital transmission, are present in *T. gondii* but absent during *H. hammondi* infections [3]. Consequently, correct differentiation of *T. gondii* oocysts from *H. hammondi* is essential for epidemiological studies and ensuring public health safety. Therefore, molecular biology techniques are employed as they allow for the rapid identification of the protozoan without performing a bioassay [19].

Berger-Schoch et al. detected small oocysts in 2 out of 252 feline fecal samples. Molecular analysis confirmed *T. gondii* in one sample and *H. hammondi* in the other. The *T. gondii*-positive case was an 11-year-old indoor individual with chronic pneumonia [38]. In our study, *T. gondii* shedding was also detected in an 18-year-old cat, which had been treated with prednisolone 1 mg/kg, twice daily (Prednicortone 20 mg tablets for dogs and cats, Dechra Regulatory B.V., Best, The Netherlands) for intestinal lymphoma. Researchers suggest that oocyst shedding and acute systemic toxoplasmosis in older cats may be associated with compromised immunity resulting from comorbidities, including infectious diseases such as Feline Immunodeficiency Virus (FIV), Feline Leukemia Virus (FeLV), Feline Infectious Peritonitis (FIP), or immunosuppressive therapy [39,40,41,42,43,44].

Diarrhea is a frequently reported symptom of *T. gondii* and other coccidian intestinal parasitosis [45,46]. Diarrhea was also reported in 2 out of 60 cats in Latvia and 6 out of 903 cats in Germany, shedding *T. gondii* oocyst [47,48]. In our research, diarrhea was observed in 80.6% (54/67) of cats shedding *T. gondii* and 33.3% (6/18) of those shedding *H. hammondi.*

*T. gondii* oocysts are rarely diagnosed via coproscopy due to the short duration of shedding. In Poland, Wąsiatycz identified *T. gondii* oocysts in 0.67% (1/149) of cat stool samples in Poznań [49]. Similarly, in Germany, coproscopic examination conducted between 2004 and 2006 in a private laboratory revealed a comparably low shedding rate (0.1%; 22/20,317). Additionally, only a few individual cats shedding oocysts were identified in studies from France, Austria, and Switzerland: 2/858, 1/994, and 1/5, respectively. In contrast, no oocyst-positive fecal samples were found in the feline population from the Netherlands (*n* = 966), Denmark (*n* = 437), or Italy (*n* = 257) [50]. In Brazil, *T. gondii* oocyst shedding was observed in 4 out of 237 stray animals (two kittens and two adult cats). Notably, these animals did not have detectable specific IgG antibodies, which suggests a primary infection [51].

The genetic diversity of *T. gondii* circulating in Europe and North America includes three major clonal lineages: type I, II, and III, which are capable of infecting humans and animals and are also commonly found in the environment (soil and water) [52,53,54,55,56,57]. Although these strains do not differ morphologically, their virulence varies significantly in experimental murine models [55]. Genotyping conducted by Lehmann et al. [58] identified more virulent atypical strains and hybrid isolates of *T. gondii*, which are significantly more prevalent in South America than in Europe or North America.

In the current study, 67 *T. gondii* DNA isolates from feline feces were genotyped using nested and multilocus PCR-RFLP. Thirteen of these isolates were identified as type I (ToxoDB#10). The genetic structure of T. *gondii* strains in cats has been analyzed across various regions globally. In Southern Thailand, among eight *T. gondii* isolates from cat feces, two were atypical, two were recombinant, one belonged to type I, two to type III, and one to either type II or III [59]. In Iran (Mashhad region), *T. gondii* type II genotype was identified in the feces of 8 out of 175 stray cats. Additionally, *T. gondii* DNA was detected in brain tissue and in the feces of 2 out of 31 deceased stray cats [60].

Numerous European studies utilizing multilocus PCR-RFLP have shown that type II is the predominant clonal lineage identified in domestic cats [38,61,62]. However, type I isolates have also been particularly reported in Spain and Italy [63,64]. In our study, type I (*T. gondii* clonal lineage I) was detected in 13 out of 67 feline fecal samples. For the first time, atypical *T. gondii* genotypes, distinct from clonal lineages I, II, and III, were identified in Germany in feces from naturally infected cats [65]. Molecular analysis of *T. gondii* strains responsible for systemic toxoplasmosis in cats also suggests genotype 3 (type II) [66]. The same genotype has also been documented in Germany [65]. In the present study, ToxoDB#3 was detected in 46 out of 67, indicating it as the dominant genotype infecting cats in Central Europe [62].

The high prevalence of this genotype may be related to its widespread occurrence in wild rodents and insectivores, which act as intermediate or paratenic hosts in the life cycle of *T. gondii* [67]. In Poland, *T. gondii* DNA, primarily genotype II and III, was detected in tissue samples from 10 wild rodents and insectivores captured in the Lublin Province [68]. In the Mazury Lake District (northeastern Poland), Grzybek et al. reported a *T. gondii* seroprevalence of 5.5% (32/577) among four rodent species [69]. However, a separate study failed to detect *T. gondii* DNA in the tissues of seropositive animals [70].

Consumption of raw or undercooked meat is considered one of the primary risk factors for *T. gondii* infection in domestic cats [71,72,73]. Genetic analysis of *T. gondii* strains isolated from sheep in Italy revealed infection with the type II clonal lineage [74]. In 19 out of 57 pork meat samples from grocery stores in the United Kingdom, *T. gondii* DNA of types II and I was detected [75]. In studies conducted in Poland by Sroka et al., *T. gondii* DNA was detected in 5.4% (175/3223) of meat product samples retailed in Poland. Genetic multilocus analysis was possible for only 61 PCR-positive samples amplified for the B1 gene. Finally, type I, type II, and type III *T. gondii* lineages were identified in 10 (10.2%), 17 (17.3%), and 48 (49.0%) samples, respectively [76]. These findings suggest that the same genotypes circulating among livestock are likely responsible for infections in domestic cats, particularly those with access to raw or undercooked meat.

## 5. Conclusions

Based on our study, the detection rate of *T. gondii* oocysts in feline feces with coproscopy methods was below 1%, and distinguishing them with *H. hammondi* oocyst without the use of molecular techniques is not feasible. The results also demonstrated considerable genetic diversity of *T. gondii* among cats across Poland. To the best of our knowledge, this is the first study confirming the presence of type I clonal lineage (ToxoDB#10) in naturally infected cats in Poland. This finding underscores the importance of implementing advanced diagnostics tools for toxoplasmosis, not only in definitive hosts, but also in susceptible species, including humans. The observed prevalence of *T. gondii* type I raises concerns regarding its ecological impact and potential risks to both human and animal health, highlighting the urgent need for further research into environmental reservoirs, transmission pathways, and the epidemiological significance of different genotypes.

## Figures and Tables

**Table 1:** List of primers used in the screening test to differentiate *Hammondia hammondi* and *Toxoplasma gondii* oocysts from feline stool samples.

Parasite Species	Forward Primer	Sequence (5′-3′)	Reverse Primer	Sequence (5′-3′)
*Hammondia hammondi*	Hham34F	ATCCCATTCCGGCTTCAGTCTTTC	Hham3R	ACAGCGGAGCCGAAGTTGGTTT
*Toxoplasma gondii*	TOX4	CGCTGCAGGGAGGAAGACGAAAGTTG	TOX5	CGCTGCAGACACAGTGCATCTGGATT

**Table 2:** List of primers and corresponding restriction enzymes used in molecular characterization of *Toxoplasma gondii* strains obtained from feline stool samples.

Markers	PCR External Primers (Sequence 5′-3′)	Nested PCR Internal Primers (Sequence 5′-3′)	Nested PCR Product	Restriction Enzymes
B1	F:TGTTCTGTCCTATCGCAACG	F:TCTTCCCAGACGTGGATTTC	530 bp	Pml1
	R:ACGGATGCAGTTCCTTTCTG	R:CTCGACAATACGCTGCTTGA		
SAG1	F:GTTCTAACCACGCACCCTGAG	F:CAATGTGCACCTGTAGGAAGC	390 bp	Sau96I and HaeII (double digest)
	R:AAGAGTGGGAGGCTCTGTGA	R:GTGGTTCTCCGTCGGTGTGAG		
5′-SAG2	F:GCTACCTCGAACAGGAACAC	F:GCATCAACAGTCTTCGTTGC	242 bp	Sau3AI
	R:GAAATGTTTCAGGTTGCTGC	R:GCAAGAGCGAACTTGAACAC		
3′-SAG2	F:TCTGTTCTCCGAAGTGACTCC	F:ATTCTCATGCCTCCGCTTC	222 bp	HhaI
	R:TCAAAGCGTGCATTATCGC	R:AACGTTTCACGAAGGCACAC		
alt. SAG2	F:GGAACGCGAACAATGAGTTT	F:ACCCATCTGCGAAGAAAACG	546 bp	HinfI and TaqI (separate digest)
	R:GCACTGTTGTCCAGGGTTTT	R:ATTTCGACCAGCGGGAGCAC		
SAG3	F:CAACTCTCACCATTCCACCC	F:TCTTGTCGGGTGTTCACTCA	225 bp	NciI
	R:GCGCGTTGTTAGACAAGACA	R:CACAAGGAGACCGAGAAGGA		
BTUB	F:TCCAAAATGAGAGAAATCGT	F:GAGGTCATCTCGGACGAACA	411 bp	BsiE and TaqI (double digest)
	R:AAATTGAAATGACGGAAGAA	R:TTGTAGGAACACCCGGACGC		
GRA6	F:ATTTGTGTTTCCGAGCAGGT	F:TTTCCGAGCAGGTGACCT	344 bp	MseI
	R:GCACCTTCGCTTGTGGTT	R:TCGCCGAAGAGTTGACATAG		
C22-8	F:TGATGCATCCATGCGTTTAT	F:TCTCTCTACGTGGACGCC	521 bp	BsmAI and MboII (double digest)
	R:CCTCCACTTCTTCGGTCTCA	R:AGGTGCTTGGATATTCGC		
C29-2	F:ACCCACTGAGCGAAAAGAAA	F:AGTTCTGCAGAGTGTCGC	446 bp	HpyCH4IV and RsaI (double digest)
	R:AGGGTCTCTTGCGCATACAT	R:TGTCTAGGAAAGAGGCGC		
L358	F:TCTCTCGACTTCGCCTCTTC	F:AGGAGGCGTAGCGCAAGT	419 bp	HaeIII and NlaIII (double digest)
	R:GCAATTTCCTCGAAGACAGG	R:CCCTCTGGCTGCAGTGCT		
PK1	F:GAAAGCTGTCCACCCTGAAA	F:CGCAAAGGGAGACAATCAGT	903 bp	AvaI and RsaI (double digest)
	R:AGAAAGCTCCGTGCAGTGAT	R:TCATCGCTGAATCTCATTGC		
Apico	F:TGGTTTTAACCCTAGATTGTGG	F:GCAAATTCTTGAATTCTCAGTT	640 bp	AflII and DdeI (double digest)
	F:AAACGGAATTAATGAGATTTGAA	R:GGGATTCGAACCCTTGATA		

**Table 3:** *Toxoplasma gondii* genotype number based on the RFLP patterns presented in ToxoDB and referenced from Brennan et al., 2016 [32] and Chen et al., 2025 [33].

Toxoplasma gondii				B1	SAG1	SAG2 5′+3′	SAG2 alt.	SAG3	BTUB	GRA6	C22-8	C29-2	L358	PK1	Apico	ToxoDB
Type I (GT1)				I	I	I	I	I	I	I	I	I	I	I	I	#10
Type II (ME49)				II/ III	II/ III	II	II	II	II	II	II	II	II	II	II	#1
Type III (CTG)				II/ III	II/ III	III	III	III	III	III	III	III	III	III	III	#2
Cats 5, 7, 8				II/ III	II/ III	II	II	II	II	II	II	II	II	II	I	#3
**Genotypes of *Toxoplasma gondii* detected in cats feces in Poland 2020–2024**																
***T. gondii* DNA isolate**	**Sex**	**Age**	**Year**	**B1**	**SAG1**	**SAG2** **5′+3′**	**SAG2** **alt.**	**SAG3**	**BTUB**	**GRA6**	**C22-8**	**C29-2**	**L358**	**PK1**	**Apico**	**ToxoDB**
Cat 1	F	5M	2020	I	I	I	I	I	I	I	I	I	I	I	I	#10
Cat 2	M	7M	2020	II/ III	II/ III	II	II	II	II	II	II	II	II	II	II	#1
Cat 3	F	4Y	2020	II/ III	II/ III	II	II	II	II	II	II	II	II	II	I	#3
Cat 4	F	5M	2020	II/ III	II/ III	II	II	II	II	II	II	II	II	II	I	#3
Cat 5	M	2Y	2020	II/ III	II/ III	II	II	II	II	II	II	II	II	II	I	#3
Cat 6	F	9M	2020	II/ III	II/ III	II	II	II	II	II	II	II	II	II	I	#3
Cat 7	M	3M	2020	II/ III	II/ III	II	II	II	II	II	II	II	II	II	I	#3
Cat 8	F	7M	2020	II/ III	II/ III	II	II	II	II	II	II	II	II	II	II	#1
Cat 9	F	1Y	2020	II/ III	II/ III	II	II	II	II	II	II	II	II	II	I	#3
Cat 10	M	18Y	2020	II/ III	II/ III	II	II	II	II	II	II	II	II	II	I	#3
Cat 11	F	8M	2020	II/ III	II/ III	II	II	II	II	II	II	II	II	II	I	#3
Cat 12	F	9M	2020	II/ III	II/ III	II	II	II	II	II	II	II	II	II	I	#3
Cat 13	M	4M	2021	I	I	I	I	I	I	I	I	I	I	I	I	#10
Cat 14	M	4M	2021	I	I	I	I	I	I	I	I	I	I	I	I	#10
Cat 15	M	3M	2021	I	I	I	I	I	I	I	I	I	I	I	I	#10
Cat 16	F	2M	2021	II/ III	II/ III	II	II	II	II	II	II	II	II	II	I	#3
Cat 17	M	11Y	2021	II/ III	II/ III	II	II	II	II	II	II	II	II	II	I	#3
Cat 18	F	3Y	2021	II/ III	II/ III	II	II	II	II	II	II	II	II	II	I	#3
Cat 19	M	10Y	2021	II/ III	II/ III	II	II	II	II	II	II	II	II	II	I	#3
Cat 20	F	8M	2022	II/ III	II/ III	II	II	II	II	II	II	II	II	II	II	#1
Cat 21	M	13Y	2022	II/ III	II/ III	II	II	II	II	II	II	II	II	II	I	#3
Cat 22	F	1Y	2022	II/ III	II/ III	II	II	II	II	II	II	II	II	II	II	#1
Cat 23	F	6M	2022	II/ III	II/ III	II	II	II	II	II	II	II	II	II	I	#3
Cat 24	M	8Y	2022	II/ III	II/ III	II	II	II	II	II	II	II	II	II	I	#3
Cat 25	M	9Y	2022	II/ III	II/ III	II	II	II	II	II	II	II	II	II	I	#3
Cat 26	F	1Y	2022	II/ III	II/ III	II	II	II	II	II	II	II	II	II	I	#3
Cat 27	M	1Y	2022	II/ III	II/ III	II	II	II	II	II	II	II	II	II	I	#3
Cat 28	M	2Y	2022	II/ III	II/ III	II	II	II	II	II	II	II	II	II	II	#1
Cat 29	M	4Y	2023	I	I	I	I	I	I	I	I	I	I	I	I	#10
Cat 30	F	7M	2023	I	I	I	I	I	I	I	I	I	I	I	I	#10
Cat 31	F	1Y	2023	I	I	I	I	I	I	I	I	I	I	I	I	#10
Cat 32	F	6M	2023	I	I	I	I	I	I	I	I	I	I	I	I	#10
Cat 33	M	13Y	2023	II/ III	II/ III	II	II	II	II	II	II	II	II	II	I	#3
Cat 34	M	2Y	2023	II/ III	II/ III	II	II	II	II	II	II	II	II	II	I	#3
Cat 35	F	4Y	2023	II/ III	II/ III	II	II	II	II	II	II	II	II	II	II	#1
Cat 36	M	8M	2023	II/ III	II/ III	II	II	II	II	II	II	II	II	II	I	#3
Cat 37	F	3Y	2023	II/ III	II/ III	II	II	II	II	II	II	II	II	II	I	#3
Cat 38	F	6Y	2023	II/ III	II/ III	II	II	II	II	II	II	II	II	II	I	#3
Cat 39	F	4M	2023	II/ III	II/ III	II	II	II	II	II	II	II	II	II	I	#3
Cat 40	M	2M	2023	II/ III	II/ III	II	II	II	II	II	II	II	II	II	I	#3
Cat 41	M	3M	2023	II/ III	II/ III	II	II	II	II	II	II	II	II	II	I	#3
Cat 42	M	16Y	2023	II/ III	II/ III	II	II	II	II	II	II	II	II	II	I	#3
Cat 43	M	11Y	2023	II/ III	II/ III	II	II	II	II	II	II	II	II	II	I	#3
Cat 44	F	2M	2023	II/ III	II/ III	II	II	II	II	II	II	II	II	II	I	#3
Cat 45	F	5M	2023	II/ III	II/ III	II	II	II	II	II	II	II	II	II	I	#3
Cat 46	M	9M	2023	II/ III	II/ III	II	II	II	II	II	II	II	II	II	I	#3
Cat 47	F	5M	2023	II/ III	II/ III	II	II	II	II	II	II	II	II	II	II	#1
Cat 48	F	6M	2024	I	I	I	I	I	I	I	I	I	I	I	I	#10
Cat 49	F	3Y	2024	II/ III	II/ III	II	II	II	II	II	II	II	II	II	I	#3
Cat 50	M	8Y	2024	II/ III	II/ III	II	II	II	II	II	II	II	II	II	I	#3
Cat 51	M	2Y	2024	II/ III	II/ III	II	II	II	II	II	II	II	II	II	I	#3
Cat 52	F	3M	2024	II/ III	II/ III	II	II	II	II	II	II	II	II	II	I	#3
Cat 53	F	9M	2024	II/ III	II/ III	II	II	II	II	II	II	II	II	II	I	#3
Cat 54	F	2Y	2024	II/ III	II/ III	II	II	II	II	II	II	II	II	II	I	#3
Cat 55	M	8M	2024	II/ III	II/ III	II	II	II	II	II	II	II	II	II	I	#3
Cat 56	M	2Y	2024	II/ III	II/ III	II	II	II	II	II	II	II	II	II	I	#3
Cat 57	M	4M	2024	I	I	I	I	I	I	I	I	I	I	I	I	#10
Cat 58	F	7M	2024	II/ III	II/ III	II	II	II	II	II	II	II	II	II	II	#1
Cat 59	F	3M	2024	II/ III	II/ III	II	II	II	II	II	II	II	II	II	I	#3
Cat 60	F	1Y	2024	II/ III	II/ III	II	II	II	II	II	II	II	II	II	I	#3
Cat 61	F	2Y	2024	II/ III	II/ III	II	II	II	II	II	II	II	II	II	I	#3
Cat 62	M	11M	2024	II/ III	II/ III	II	II	II	II	II	II	II	II	II	I	#3
Cat 63	F	4M	2024	I	I	I	I	I	I	I	I	I	I	I	I	#10
Cat 64	F	6M	2024	I	I	I	I	I	I	I	I	I	I	I	I	#10
Cat 65	F	6M	2024	I	I	I	I	I	I	I	I	I	I	I	I	#10
Cat 66	M	7M	2024	II/ III	II/ III	II	II	II	II	II	II	II	II	II	I	#3
Cat 67	M	7M	2024	II/ III	II/ III	II	II	II	II	II	II	II	II	II	I	#3

**Table 4:** Annual distribution of feline stool samples testing positive for *T. gondii* and *H. hammondi* in Poland.

	Examined Feline Stool Samples	*Toxoplasma gondii* Positive Samples		*Hammondia hammondi* Positive Samples	
Year	*n*	*n*	%	*n*	%
2020	1780	12	0.67	1	0.06
2021	5760	7	0.12	6	0.10
2022	11,641	9	0.08	5	0.04
2023	19,121	19	0.10	4	0.02
2024	23,346	20	0.09	2	0.01
Total:	61,648	67	0.11	18	0.03

**Table 5:** Comparison of *T. gondii* and *H. hammondi* positive samples according to host age, sex, stool consistency, co-infections, and identified clonal lineage.

	*T. gondii*		*H. hammondi*	
	Age <1 Y	Age ≥1	Age <1	Age ≥1
Total	37	30	8	10
Male	14	17	5	4
Female	23	13	3	6
Diarrhea	36	18	5	1
Formed stool	1	12	3	9
co-infection	9	5	1	2
*Giardia duodenalis*	4	1	0	1
*Cystoisospora felis*	2	3	0	0
*Tritrichomonas foetus*	1	1	1	1
*Toxocara cati*	2	0	0	0
no co-infection	28	25	7	8
*T. gondii* clonal lineage I	11	2	-	-
*T. gondii* clonal lineage II	26	28	-	-

## Data Availability

The original contributions presented in this study are included in the article. Further inquiries can be directed to the corresponding author(s).
